# Cross-Modal Assessment of Post-Cholecystectomy Symptoms: Integrating MRCP Metrics with Upper Endoscopy

**DOI:** 10.3390/tomography12030039

**Published:** 2026-03-16

**Authors:** Davut Unsal Capkan, Ibrahim Tayfun Sahiner

**Affiliations:** 1 Department of Radiology, Medical Point Hospital, 27060 Gaziantep, Turkey; 2 Department of General Surgery, Medical Point Hospital, 27060 Gaziantep, Turkey; tayfunsahiner@gmail.com

**Keywords:** post-cholecystectomy syndrome, MRCP, common bile duct diameter, endoscopy, oddi dysfunction

## Abstract

Background/Objectives: Post-cholecystectomy syndrome (PCS) remains diagnostically challenging due to overlapping biliary and non-biliary causes. This study aimed to evaluate whether common bile duct (CBD) diameter measured by MRCP can serve as a practical triage parameter in symptomatic PCS patients and to define a data-supported threshold for predicting clinically relevant biliary pathology. Secondary objectives included assessing correlations between MRCP findings and upper endoscopic features. Methods: In this retrospective single-center study, symptomatic adults undergoing upper endoscopy and MRCP were analyzed. Demographic, clinical, biochemical, radiologic, and endoscopic variables were recorded. Diagnostic performance was assessed using ROC analysis, and independent predictors of biliary dilatation were evaluated with multivariable logistic regression. Results: We analyzed 141 symptomatic post-cholecystectomy patients (mean age 58.2 ± 16.3 years; 67.4% female; median time since surgery 18 [9–36] months). Major symptoms: abdominal pain 84.9%, dyspepsia/bloating 47.5%, nausea/vomiting 22.3%, diarrhea 15.1%. CBD diameter measurements were available in the MRCP subgroup (n = 45); ERCP was performed selectively (n = 12). MRCP findings: CBD ≥ 7 mm 31.9%, biliary dilatation 14.9%, stricture 2.8%, suspected Oddi dysfunction 11.3%, postoperative complications 39.7%. Endoscopy: mucosal inflammation 91.5%; normal 8.5%. Significant correlations included CBD diameter vs. mucosal inflammation (r = 0.32, *p* = 0.001), dilatation vs. bile reflux (r = 0.28, *p* = 0.004), and Oddi dysfunction vs. papillary edema (r = 0.41, *p* = 0.001). CBD diameter showed the best diagnostic performance (AUC 0.82, 95% CI 0.74–0.90; cut-off ≥ 8.0 mm; sensitivity 78.3%; specificity 81.5%; *p* < 0.001). In multivariable analysis, age independently predicted biliary dilatation (OR 1.05 per year; 95% CI 1.01–1.09; *p* = 0.007). Conclusions: In symptomatic post-cholecystectomy patients, MRCP-measured CBD diameter provides a useful metric for risk stratification, with a threshold of ≥8 mm identifying patients more likely to harbor biliary pathology. These findings support a structured diagnostic approach that prioritizes noninvasive imaging while reserving ERCP for selected cases. Further prospective validation is warranted.

## Simple Summary

Some patients continue to experience abdominal pain, bloating, or digestive discomfort after gallbladder removal, a condition known as post-cholecystectomy syndrome. Determining whether these symptoms are caused by bile duct problems or by other gastrointestinal conditions can be challenging. In this study, we investigated whether measuring the diameter of the common bile duct using a noninvasive imaging method called magnetic resonance cholangiopancreatography (MRCP) could help identify patients who are more likely to have significant biliary abnormalities. We found that a bile duct diameter of 8 mm or greater was associated with a higher likelihood of underlying biliary pathology. While endoscopy frequently revealed mucosal inflammation, bile duct size provided stronger risk stratification for structural biliary problems. These findings suggest that careful interpretation of MRCP measurements may support a structured and less invasive diagnostic approach, reserving more invasive procedures such as ERCP for selected patients. Further prospective studies are needed to confirm these results.

## 1. Introduction

Cholecystectomy reliably alleviates symptoms in most patients with gallstone disease, yet a clinically relevant subset continues to experience persistent or de novo upper abdominal complaints after surgery. This constellation—commonly termed post-cholecystectomy syndrome (PCS)—encompasses biliary colic or right upper quadrant pain together with nonspecific gastrointestinal symptoms such as dyspepsia, bloating, and altered bowel habits [1]. Reported rates of persistent pain vary across cohorts and methodologies, but systematic reviews indicate that upper abdominal pain may persist in up to one-third of patients and arise de novo in approximately one in seven, while diarrhea and constipation are among the most frequent ongoing symptoms [2,3,4]. Earlier prospective work similarly documented a nontrivial burden of persistent pain after laparoscopic cholecystectomy and highlighted preoperative symptom profiles associated with poorer postoperative outcomes [5].

The etiological spectrum of PCS is broad and spans biliary and non-biliary conditions. Biliary causes include retained or recurrent common bile duct (CBD) stones, biliary strictures or leaks, papillary dysfunction, and functional disorders of the biliary tract; non-biliary contributors range from peptic disease to functional gastrointestinal disorders [6,7]. Among biliary causes, dysfunction of the biliary sphincter of Oddi (SOD) is a prominent consideration in patients with biliary-type pain, particularly after gallbladder removal; contemporary reviews and the Rome IV framework categorize functional biliary disorders and clarify diagnostic criteria, while acknowledging ongoing gaps in evidence and the need for careful selection for invasive testing [8,9,10].

Imaging plays a central role in the evaluation of post-cholecystectomy symptoms. Magnetic resonance cholangiopancreatography (MRCP) is a noninvasive technique with high accuracy for detecting choledocholithiasis and delineating biliary anatomy and is often preferred as an initial advanced imaging modality [11,12,13,14]. Meta-analytic and large cohort data have reported MRCP sensitivities commonly in the 90% range for CBD stones, with excellent negative predictive value, supporting its use to triage patients and reduce unnecessary invasive procedures [14,15,16]. ERCP, in contrast, provides both diagnostic and therapeutic capabilities—allowing stone extraction, stricture dilation, and sphincter therapy—but carries procedure-related risks; accordingly, contemporary practice favors reserving ERCP for cases with positive noninvasive findings or high pretest probability of obstructive pathology [17,18,19]. Cross-sectional CT can complement MRCP in characterizing strictures and mass-like lesions, while radiology-pathology correlation remains essential when malignancy is suspected [20,21].

Despite these advances, optimal sequencing and integration of endoscopic and radiologic assessments in suspected PCS remains unsettled, especially in real-world populations where coexisting functional disorders are common. Radiology literature emphasizes detection of postoperative complications (e.g., bile duct injury, retained stones), whereas endoscopic reports may better capture mucosal inflammation and papillary dysfunction that contribute to symptomatology [20]. Robust comparative data that correlate MRCP (and other imaging) with endoscopic findings—including mucosal disease, papillary appearance, and therapeutic outcomes—are comparatively limited, and existing studies are heterogeneous in inclusion criteria and reference standards [4,8,11].

In contemporary practice, additional modalities such as endoscopic ultrasound (EUS) and hepatobiliary scintigraphy may contribute to the evaluation of microlithiasis, distal CBD pathology, and functional biliary obstruction when cross-sectional imaging is inconclusive. Accordingly, the primary aim of the present study was to evaluate whether CBD diameter measured on MRCP can function as a practical triage parameter in symptomatic post-cholecystectomy patients and to define an empirically supported cut-off value for predicting clinically relevant biliary pathology. Secondary aims included quantifying cross-modal correlations between MRCP-based structural findings and contemporaneous upper endoscopic abnormalities to better contextualize symptom drivers and refine diagnostic sequencing.

## 2. Materials and Methods

### 2.1. Study Design and Ethical Approval

This retrospective descriptive study was conducted at Medical Point Hospital, Department of Gastroenterology, between January 2023 and December 2025. The study included patients who had undergone cholecystectomy and later presented with upper abdominal pain, dyspepsia, bloating, or diarrhea suggestive of PCS. For the purposes of this study, PCS was defined clinically as persistent or new-onset upper abdominal symptoms following cholecystectomy in the absence of alternative acute surgical pathology at the time of evaluation. The research protocol was approved by the Gaziantep City Hospital Non-Interventional Clinical Research Ethics Committee (Decision No: 2025/220, Date: 21 May 2025), and the study adhered to the principles of the Declaration of Helsinki.

### 2.2. Study Population

**Figure 1:**
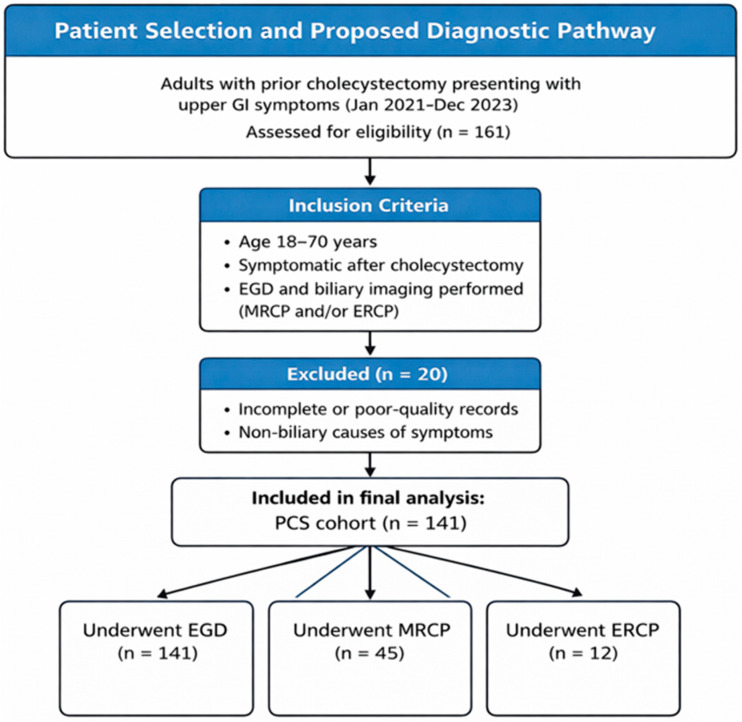
Patient Selection Flowchart.

Eligible participants were adults aged 18 to 70 years who had previously undergone cholecystectomy and were evaluated at any point after surgery for gastrointestinal symptoms such as upper abdominal pain, dyspepsia, or bloating. Inclusion required upper endoscopy, with MRCP performed when clinically indicated and complete analyzable records. ERCP, when performed, was used for therapeutic purposes. Patients were excluded if they had incomplete or non-assessable imaging data, a history of biliary or pancreatic malignancy, or evidence that their symptoms were due to non-biliary gastrointestinal disorders. Additional exclusion criteria included age below 18 or above 70 years, pregnancy, or belonging to legally or medically restricted groups (such as prisoners or individuals with cognitive impairment). The chosen age range ensured inclusion of an adult population with optimal imaging quality and minimized the influence of age-related comorbidities that could confound diagnostic interpretation. Before labeling symptoms as post-cholecystectomy syndrome, alternative non-biliary causes were excluded based on clinical assessment, laboratory testing, and upper gastrointestinal endoscopy. Specifically, peptic ulcer disease, erosive gastritis, active gastrointestinal bleeding, and other identifiable luminal gastrointestinal pathologies explaining the symptoms were ruled out. Routine preoperative upper gastrointestinal endoscopy was not systematically available for all patients, as this retrospective cohort was derived from routine clinical practice. Therefore, the presence of pre-existing mucosal pathology prior to cholecystectomy could not be definitively excluded in all cases (Figure 1).

### 2.3. Radiologic Evaluation

MRCP was requested at the discretion of the treating clinician for persistent biliary-type symptoms, abnormal liver function tests, or inconclusive prior imaging. This practice may introduce selection bias, which is acknowledged as a limitation of the study. Magnetic resonance cholangiopancreatography (MRCP) was performed using a 1.5-Tesla MRI system with standard heavily T2-weighted coronal and axial sequences optimized for detailed visualization of the biliary tree. The following parameters were evaluated: CBD diameter (with a value of ≥ 7 mm considered dilated), intrahepatic biliary dilatation, presence of retained or recurrent stones, postoperative complications such as biloma or localized fluid collections, and biliary strictures indicating ductal narrowing. A CBD diameter ≥7 mm was considered dilated, in line with commonly used imaging thresholds in post-cholecystectomy populations, while acknowledging that no absolute cut-off exists and that ductal caliber may increase with age and prior cholecystectomy [11,12,20].

When MRCP suggested obstructive pathology or when therapeutic intervention was required, ERCP was performed as a therapeutic procedure (e.g., sphincterotomy/stone extraction/stenting). During ERCP, the morphology of the papilla, ease of cannulation, and the presence of choledocholithiasis, sludge, or strictures were carefully recorded. Therapeutic procedures, including sphincterotomy, balloon extraction, or stent placement, were documented when performed. MRCP images were interpreted by an experienced radiologist blinded to endoscopic findings. CBD diameter was measured on T2-weighted images using the maximal inner-to-inner ductal diameter, preferentially on axial sections and confirmed on coronal reformats when available.

CBD dilatation was defined as ≥7 mm on MRCP, consistent with commonly used post-cholecystectomy imaging thresholds, acknowledging potential age-related variation. For ROC analysis, CBD diameter was treated as a continuous variable, and the optimal diagnostic cut-off was determined using the Youden index.

Measurements were obtained at the mid-extrahepatic bile duct level, avoiding the intrapancreatic segment to minimize obliquity and partial volume effects. All measurements were performed using the hospital PACS tools (Centricity PACS; GE Healthcare, Chicago, IL, USA) according to a standardized protocol. Because this was a retrospective study, formal interobserver variability analysis was not performed; however, all images were re-reviewed by the study radiologist to ensure internal consistency.

Suspected sphincter of Oddi dysfunction on MRCP was operationally defined as distal CBD tapering or delayed emptying in the absence of a visible obstructive lesion (stone, stricture, or mass), interpreted in conjunction with clinical findings.

### 2.4. Endoscopic Evaluation

All patients underwent diagnostic upper gastrointestinal endoscopy (esophagogastroduodenoscopy, EGD) under conscious sedation, using a standard video-endoscope system. The endoscopic findings were systematically recorded and categorized as normal or abnormal. Abnormal findings included bile reflux, gastric or duodenal erythema, erosions, ulcerations, and papillary abnormalities such as edema, hyperemia, or anatomic deformity. These findings were interpreted as potential indicators of altered bile flow or sphincter dysfunction following cholecystectomy.

Endoscopic reports were obtained from the hospital’s digital archive and reviewed by two investigators to ensure consistency. The morphology and functional appearance of the Papilla Vateri were specifically assessed in all available cases, noting any structural variations or signs of dysfunction. The presence of mucosal inflammation (gastritis, duodenitis, or ulcer disease) was also documented and compared with the radiologic findings to explore potential correlations between structural and mucosal changes. All upper endoscopic procedures were performed by experienced gastroenterologists. Endoscopic findings were reviewed independently by two investigators blinded to radiologic results; discrepancies were resolved by consensus.

Endoscopic mucosal inflammation was defined according to macroscopic criteria documented in routine clinical reporting. When available, gastritis was graded in accordance with the Updated Sydney System (topographic distribution and severity of erythema, erosions, or ulceration), and esophagitis was classified using the Los Angeles (LA) classification system [22,23]. In cases where histopathology was performed, biopsy results—including assessment for Helicobacter pylori—were recorded; however, biopsy sampling was not mandatory in all patients due to the retrospective design.

Papillary edema/hyperemia was defined as visible swelling, erythema, or congestion of the major duodenal papilla during EGD, as documented in endoscopic reports. “Signs of SOD” were defined descriptively based on endoscopic impression of papillary abnormality (edema, deformity, or difficult cannulation during ERCP when performed), in conjunction with radiologic suspicion of distal CBD narrowing or functional obstruction. Formal manometric confirmation was not available.

### 2.5. Evaluated Parameters

The primary, a priori objective was to determine the diagnostic performance of MRCP-measured CBD diameter for predicting clinically significant biliary abnormalities and to establish an optimal threshold using ROC analysis. Secondary objectives included (i) quantifying the prevalence of MRCP-defined biliary abnormalities, and (ii) assessing prespecified correlations between MRCP metrics and endoscopic findings (mucosal inflammation, bile reflux, papillary changes). Multivariable modeling was performed to identify independent predictors of biliary dilatation. Multivariable modeling was prespecified to identify predictors of biliary dilatation, limited to clinically relevant covariates to minimize overfitting. All data were retrospectively retrieved from patients’ electronic medical records and the hospital’s digital imaging and endoscopy archive. The following parameters were systematically evaluated to characterize biliary and gastrointestinal findings: CBD diameter and the presence of biliary dilatation; residual or recurrent stones detected on MRCP and/or ERCP; biliary strictures or postoperative complications such as biloma, localized fluid collection, or infection; findings indicative of Oddi sphincter dysfunction; mucosal changes including gastritis, duodenitis, or ulceration observed during endoscopy; and morphologic or functional alterations of the Papilla Vateri, such as edema, hyperemia, or structural deformity. Additionally, clinical and biochemical data were recorded for each patient, including age, sex, time elapsed since cholecystectomy, and presenting symptoms (upper abdominal pain, dyspepsia, bloating, or diarrhea). Time since cholecystectomy was recorded in months; analyses treated timing as a continuous descriptive variable rather than stratifying by postoperative day. Laboratory variables such as alanine aminotransferase (ALT), aspartate aminotransferase (AST), alkaline phosphatase (ALP), and total bilirubin levels were also collected to support clinical and imaging correlations. Time since cholecystectomy was recorded in months; analyses treated timing as a continuous descriptive variable rather than stratifying by postoperative day.

### 2.6. Statistical Analysis

All statistical analyses were performed using IBM SPSS Statistics version 27.0 (IBM Corp., Armonk, NY, USA). Continuous variables were presented as mean ± standard deviation (SD) or median (interquartile range, IQR), while categorical variables were expressed as numbers and percentages. Comparisons between different radiologic or endoscopic parameters were made using the independent samples *t*-test for continuous data and the Chi-square test for categorical variables. The Receiver Operating Characteristic (ROC) curve analysis was conducted to assess the diagnostic performance of radiologic parameters—particularly CBD diameter—in predicting clinically significant biliary pathology among PCS patients. The area under the curve (AUC) was calculated, and the optimal cutoff value was determined using the Youden index. To identify independent predictors of abnormal endoscopic or radiologic findings, a multivariate logistic regression analysis was performed. Correlations between radiologic and endoscopic parameters were evaluated using the Pearson or Spearman correlation test, depending on data distribution. Given the exploratory nature and the limited number of events for some outcomes, the number of tested associations was prespecified and restricted. The multivariable model was limited to a small set of clinically relevant covariates to reduce overfitting; findings should be interpreted accordingly. A *p*-value of <0.05 was considered statistically significant for all analyses.

## 3. Results

**Figure 2:**
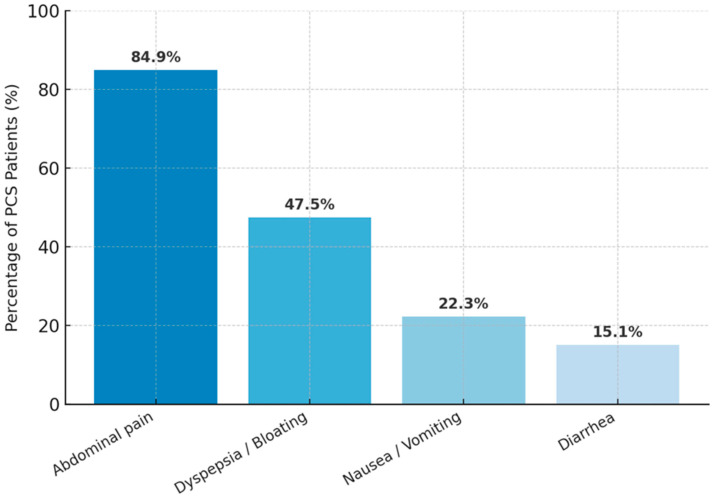
Frequency of major gastrointestinal symptoms among PCS patients (n = 141).

A total of 141 patients with post-cholecystectomy syndrome were included in the study. The mean age of the patients was 58.2 ± 16.3 years, and 67.4% were female (95 females and 46 males). The median interval from cholecystectomy to symptom onset was 12 months [IQR: 6–24], and the median interval from symptom onset to MRCP/EGD evaluation was 4 months [IQR: 2–8]. The median total time from cholecystectomy to diagnostic evaluation (MRCP/EGD) was 18 months [IQR: 9–36]. No patient underwent routine ERCP; ERCP (n = 12) was performed selectively based on clinical/imaging indications, and CBD diameter was available in the MRCP subgroup (n = 45). The most common presenting symptom was abdominal pain, observed in 120 patients (84.9%), followed by dyspepsia or bloating in 67 patients (47.5%), nausea or vomiting in 32 patients (22.3%), and diarrhea in 21 patients (15.1%). Laboratory findings showed that the mean alanine aminotransferase (ALT) level was 41.2 ± 19.6 U/L, the mean aspartate aminotransferase (AST) level was 39.8 ± 17.2 U/L, the mean alkaline phosphatase (ALP) level was 122.4 ± 58.7 U/L, and the mean total bilirubin level was 0.88 ± 0.34 mg/dL (Table 1, Figure 2).

**Figure 3:**
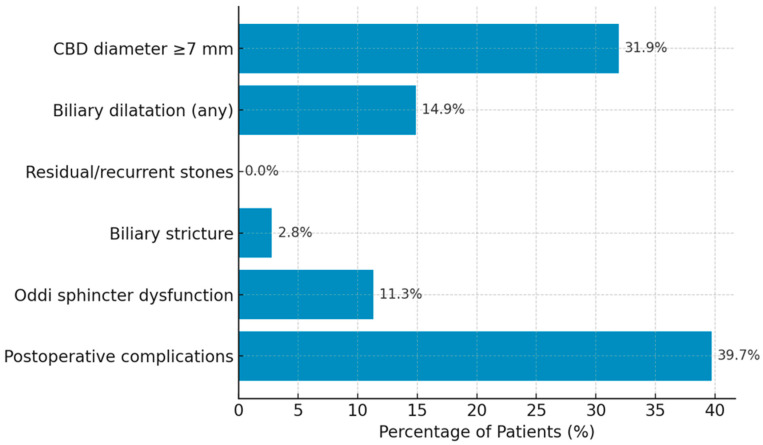
Distribution of Radiologic Findings (n = 141).

Radiologic metrics reported here derive primarily from MRCP. Radiologic evaluation revealed that 45 patients (31.9%) had a CBD diameter of ≥7 mm, indicating ductal dilatation. Biliary dilatation of any degree was present in 21 patients (14.9%), while biliary stricture was detected in 4 patients (2.8%). Findings suggestive of Oddi sphincter dysfunction were observed in 16 patients (11.3%). Postoperative radiologic complications, including biloma, abscess, or localized fluid collections, were identified in 56 patients (39.7%) (Table 2, Figure 3).

**Figure 4:**
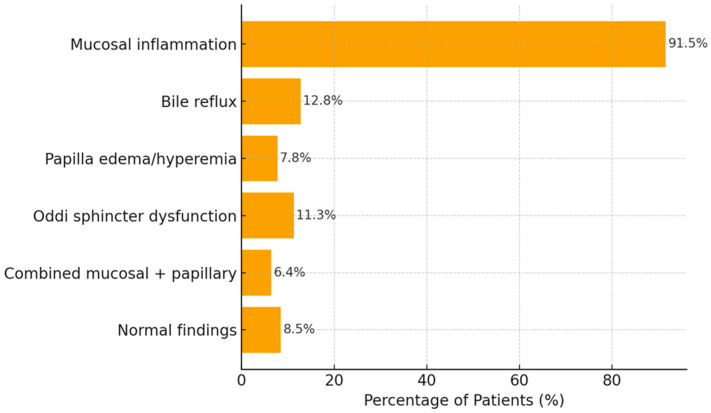
Distribution of Endoscopic Findings (n = 141).

Endoscopic evaluation showed that mucosal inflammation, including gastritis, duodenitis, or ulceration, was present in 129 patients (91.5%). Bile reflux was observed in 18 patients (12.8%), while papilla Vateri edema or hyperemia was detected in 11 patients (7.8%). Signs of Oddi sphincter dysfunction on endoscopy were identified in 16 patients (11.3%). Combined mucosal and papillary pathology was present in 9 patients (6.4%). Normal endoscopic findings were reported in 12 patients (8.5%) (Table 3, Figure 4).

Correlation analysis demonstrated a mild positive relationship between CBD diameter and mucosal inflammation (r = 0.32, *p* = 0.001). A significant correlation was also found between biliary dilatation and bile reflux (r = 0.28, *p* = 0.004). Oddi sphincter dysfunction was moderately correlated with papillary edema or hyperemia (r = 0.41, *p* = 0.001). A weak but statistically significant correlation was observed between biliary stricture and papillary deformity (r = 0.22, *p* = 0.03). The association between postoperative complications and mucosal inflammation showed a borderline significance (r = 0.18, *p* = 0.06) (Table 4).

**Figure 5:**
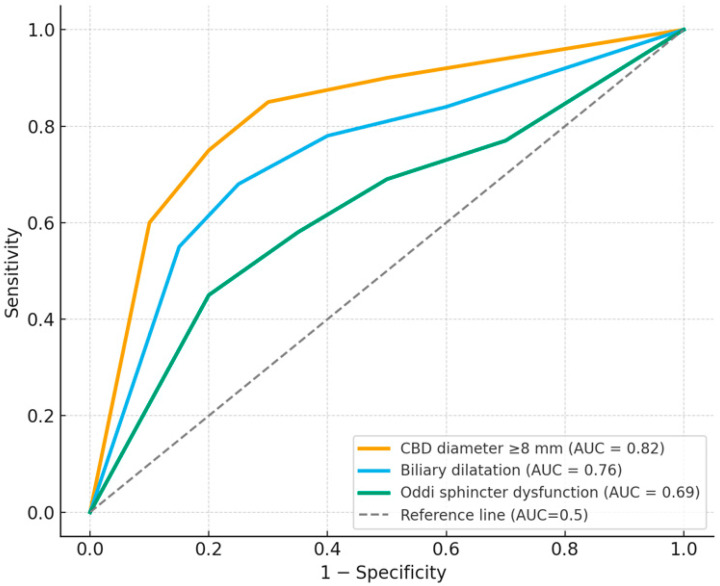
ROC Curves for MRCP-Based Predictors of Biliary Dilatation/Abnormal Biliary Findings.

ROC curve analysis demonstrated that CBD diameter had the highest diagnostic accuracy for predicting biliary abnormalities, with an AUC of 0.82 (95% CI: 0.74–0.90, *p* < 0.001) and an optimal cut-off value of ≥8.0 mm, providing 78.3% sensitivity and 81.5% specificity. Biliary dilatation of any degree also showed good diagnostic performance (AUC = 0.76, 95% CI: 0.67–0.85, *p* = 0.002), with 72.4% sensitivity and 70.3% specificity. Oddi sphincter dysfunction had a moderate predictive value (AUC = 0.69, 95% CI: 0.58–0.80, *p* = 0.011). Biliary stricture demonstrated a lower discriminative power (AUC = 0.62, 95% CI: 0.51–0.73, *p* = 0.046) (Table 5, Figure 5).

In the multivariate logistic regression analysis, increasing age was identified as an independent predictor of biliary dilatation (*OR* = 1.05 per year; 95% CI: 1.01–1.09; *p* = 0.007). Although biliary stricture showed a high odds ratio (*OR* = 9.12; 95% CI: 0.78–106.08), this association did not reach statistical significance (*p* = 0.077), likely due to the small number of cases with strictures. Neither Oddi sphincter dysfunction (*p* = 0.359) nor postoperative complications (*p* = 0.994) were independently associated with biliary dilatation (Table 6).

## 4. Discussion

This study evaluated whether MRCP-measured CBD diameter can function as a practical triage parameter in symptomatic post-cholecystectomy patients. Our findings indicate that ductal caliber was the most discriminative radiologic parameter within this cohort for identifying biliary pathology, with a threshold of ≥8 mm demonstrating robust diagnostic performance. In contrast, correlations between structural MRCP findings and endoscopic abnormalities were generally modest, underscoring that while mucosal and papillary changes are common, they do not provide the same level of stratification value as ductal measurements.

Our cohort’s symptom profile—abdominal pain predominating, followed by dyspepsia/bloating—mirrors prior syntheses reporting substantial rates of persistent or de novo pain and dyspeptic complaints after cholecystectomy [2,4]. Practical guidance emphasizes a broad differential that spans biliary and non-biliary drivers [4], consistent with the dual structural–mucosal signal we observed.

The moderate correlation between endoscopic papillary changes and suspected Oddi dysfunction fits contemporary descriptions of functional biliary disorders under Rome IV [9,10] and the clinical ambiguity highlighted by Kegnæs et al.—namely overlapping symptomatology, imperfect noninvasive markers, and the need to avoid indiscriminate invasive testing [8]. Our finding that Oddi dysfunction was not an independent predictor of ductal dilatation in multivariable modeling supports the view that papillary/functional abnormalities contribute variably to the structural phenotype and should be interpreted in context rather than used as solitary triggers for ERCP [8,9,10]. The high prevalence of mucosal inflammation in our cohort likely reflects the broad and pragmatic endoscopic categorization used in routine clinical practice. This category may encompass nonspecific gastritis, bile reflux-related changes, and age-related incidental findings that are common in older populations. Although statistically significant correlations with CBD diameter were observed, their clinical magnitude should be interpreted cautiously.

These findings are consistent with prior meta-analytic and comparative studies that establish MRCP as a high-accuracy modality for evaluating ductal pathology [11,14,15,16]. In this context, ductal measurements provide objective structural information that may complement clinical assessment and guide further diagnostic steps. Given the procedural risks associated with ERCP, invasive intervention should remain indication-driven rather than routine, particularly in the absence of clear obstructive findings. It should be emphasized that diagnostic performance metrics, including ROC-derived thresholds, were calculated within the MRCP subgroup (n = 45). As such, these estimates may be subject to spectrum bias and should not be interpreted as universally generalizable across the broader PCS population. The identified ≥8 mm threshold should therefore be viewed as exploratory and context-dependent rather than definitive.

It is important to recognize that comprehensive contemporary evaluation of PCS extends beyond MRCP and upper endoscopy alone. EUS has been shown to provide high sensitivity for detecting microlithiasis, small retained stones, distal CBD lesions, and ampullary pathology that may not be visualized on MRCP, particularly in patients with persistent biliary-type symptoms [18,21]. In addition, hepatobiliary scintigraphy remains a noninvasive method for functional assessment of biliary drainage and may contribute to the evaluation of suspected sphincter of Oddi dysfunction within the broader Rome IV framework [8,9]. These modalities were not systematically incorporated in our retrospective cohort. Therefore, the proposed diagnostic framework should be interpreted as an imaging-centered pathway derived from available data rather than a complete representation of all real-world diagnostic strategies in PCS.

In contemporary clinical practice, evaluation of PCS is typically multidisciplinary and stepwise, incorporating symptom characterization, laboratory testing, transabdominal ultrasonography, cross-sectional imaging, EUS when microlithiasis or distal pathology is suspected, and functional testing in selected cases. Our study does not attempt to redefine this comprehensive pathway; rather, it examines how MRCP-derived ductal measurements relate to endoscopic findings within the subset of imaged patients. Therefore, the present analysis should be interpreted as contributing one structural component within a broader diagnostic framework rather than representing a complete clinical algorithm.

It is important to acknowledge that the definition of post-cholecystectomy CBD dilatation remains controversial. Several studies have reported that CBD diameter may increase physiologically after cholecystectomy, with proposed upper limits ranging from 7 mm to 10 mm depending on age, imaging modality, and population characteristics [11,20]. Age-related ductal enlargement and adaptive remodeling following gallbladder removal further complicate interpretation of a single numerical threshold [9,20]. Therefore, the ≥7 mm definition used in our study reflects a commonly applied imaging reference rather than an absolute pathological boundary. Importantly, the ≥8 mm cut-off derived from ROC analysis in this symptomatic cohort may assist in risk stratification within similar clinical settings and should not be interpreted as a universal definition of abnormality. Clinical context and associated findings remain essential in interpreting ductal caliber.

Although we observed a large odds ratio linking stricture with dilatation, the association was not statistically significant—likely a function of low stricture frequency and wide confidence intervals. This pattern is compatible with imaging-focused literature showing that while strictures often co-occur with dilated ducts, they do not consistently emerge as independent predictors once other factors are modeled [20,21]. In clinical terms, strictures signal a relevant subset requiring targeted therapy, but they may not drive population-level prediction.

An additional noteworthy finding was the relatively high proportion of radiologically detected postoperative complications (39.7%), including biloma, localized collections, and suspected infectious changes. While not all such findings were clinically significant, their presence underscores the heterogeneity of PCS presentations and highlights the importance of comprehensive imaging assessment. Management was individualized according to imaging and clinical severity, ranging from conservative monitoring and antibiotic therapy in stable cases to percutaneous drainage or endoscopic intervention when indicated. Although postoperative complications were not independent predictors of biliary dilatation in multivariable analysis, their detection remains clinically relevant, as early identification may prevent progression to more severe outcomes.

The inclusion of postoperative complications within the PCS framework warrants clarification. PCS is widely described as a heterogeneous clinical entity encompassing both biliary and non-biliary causes of persistent or new-onset symptoms following cholecystectomy [24,25]. Persistent symptoms may arise from functional disorders, retained stones, ductal injury, or other postoperative sequelae, reflecting the broad etiological spectrum of PCS [5]. Management-oriented studies comparing laparoscopic and endoscopic strategies for choledocholithiasis further illustrate that postoperative biliary findings and complications may overlap clinically with PCS presentations [26,27]. In addition, adaptive ductal enlargement following cholecystectomy has been documented, complicating strict diameter-based definitions of abnormality [28].

Within this broader clinical context, early or delayed postoperative complications—such as biloma, localized collections, or bile duct injury—may manifest with PCS-type symptoms, even though their underlying mechanisms differ from functional biliary disorders. We therefore considered radiologically detected postoperative complications within the symptomatic PCS spectrum, as they represent clinically relevant causes of post-cholecystectomy complaints. However, some authors advocate for a narrower definition of PCS limited to functional or obstructive etiologies, distinguishing it from discrete surgical complications [24,25]. This conceptual variability should be acknowledged when interpreting prevalence estimates and comparing studies.

From a practical perspective, ductal caliber can assist in stratifying patients who may benefit from further biliary evaluation. In contrast, the high prevalence of mucosal abnormalities highlights the importance of complementary endoscopic assessment. Given the procedural risks associated with ERCP, a stepwise evaluation strategy remains appropriate in symptomatic patients [11,12,14,19].

Prospective studies integrating standardized symptom scales, quantitative MRCP metrics (e.g., ductal caliber indexed to age/body size), and functional testing (manometry or secretin-MRCP) could refine thresholds and disentangle causal pathways in PCS. Prospective comparative studies would be valuable to assess whether MRCP-informed diagnostic sequencing influences procedure rates, diagnostic timelines, and patient-centered outcomes.

Compared with prior reports that have primarily focused on imaging accuracy (e.g., MRCP versus ERCP for choledocholithiasis) or symptom-oriented clinical guidance without systematic cross-modal linkage [4,8,12,14,20], our study explores the relationship between MRCP findings and contemporaneous upper endoscopic observations within a symptomatic PCS cohort. Rather than proposing a fundamentally new diagnostic construct, these findings provide exploratory data on cross-modal concordance (e.g., ductal caliber and mucosal changes) and suggest that structural ductal metrics may contribute to structured clinical assessment when interpreted alongside endoscopic findings [4,9,10]. These observations should be viewed as complementary to the existing literature on MRCP performance and ERCP selectivity [11,12,14] rather than as a replacement for comprehensive multimodal evaluation strategies.

This study has some limitations. First, its retrospective, single-center design may limit generalizability and introduce selection bias. Second, sphincter of Oddi dysfunction was not confirmed by manometry, and classification relied on descriptive endoscopic and radiologic impressions, which may have led to misclassification. Formal interobserver variability analysis for CBD measurements was not performed, which may affect measurement reproducibility. Third, the low number of biliary stricture events resulted in wide confidence intervals and limited statistical precision. Fourth, the absence of a non-symptomatic comparator group precludes assessment of background rates of ductal dilatation or mucosal abnormalities in asymptomatic post-cholecystectomy patients. Finally, the very high prevalence of mucosal inflammation reduced multivariable separability for this variable and therefore it was not retained in the primary adjusted model. An additional limitation relates to the absence of systematic preoperative endoscopic evaluation. Although alternative luminal causes were excluded at the time of post-cholecystectomy assessment, we cannot exclude the possibility that some mucosal abnormalities detected on EGD represented pre-existing gastrointestinal pathology rather than de novo PCS-related changes. Furthermore, EUS and functional biliary testing were not systematically incorporated into this retrospective cohort, although these modalities play an important role in contemporary PCS evaluation, particularly when MRCP findings are inconclusive.

Structural and mucosal abnormalities were frequently observed in this cohort; however, ductal measurements provided the most consistent stratification signal for biliary pathology. Age-related ductal enlargement likely contributed to observed variation, supporting the need for contextual interpretation of CBD measurements in post-cholecystectomy patients.

**Figure 6:**
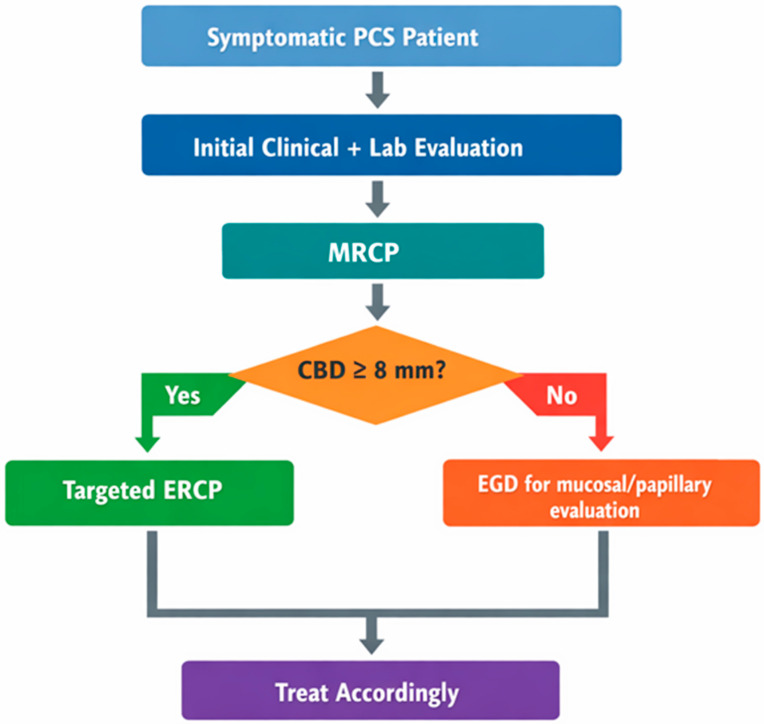
MRCP-Informed Component within PCS Evaluation. Exploratory framework derived from retrospective cohort.

Overall, our findings suggested that an MRCP-informed component may be incorporated into structured diagnostic frameworks in similar clinical contexts. A schematic representation of this proposed pathway is provided in Figure 6. Prospective multicenter studies incorporating standardized symptom scales, quantitative MRCP metrics, and functional testing will be essential to validate these findings and refine patient-centered triage strategies.

## 5. Conclusions

In conclusion, in symptomatic post-cholecystectomy patients, MRCP-measured CBD diameter may assist as a radiologic metric for biliary risk stratification, with an empirically derived threshold of ≥8 mm observed in this cohort. Age was independently associated with biliary dilatation, consistent with established knowledge of age-related ductal widening. In contrast, biliary stricture showed an imprecise association, and neither suspected sphincter of Oddi dysfunction nor postoperative complications independently predicted ductal enlargement. These findings suggest that an MRCP-informed diagnostic approach may be reasonable in similar clinical settings, in which noninvasive imaging guides subsequent evaluation and ERCP is reserved for clearly defined indications.

## Figures and Tables

**Table 1:** Baseline Characteristics of the Study Population.

Variables	PCS Group (n = 141)
	Mean ± SD or Median [IQR] or n (%)
Age (years)	58.2 ± 16.3
Gender (Female/Male)	95/46
Interval from cholecystectomy to symptom onset (months)	12 [6–24]
Interval from symptom onset to MRCP/EGD (months)	4 [2–8]
Median time from cholecystectomy to evaluation (MRCP/EGD), months	18 [9–36]
Major presenting symptom	
Abdominal pain	120 (84.9%)
Dyspepsia/Bloating	67 (47.5%)
Nausea/Vomiting	32 (22.3%)
Diarrhea	21 (15.1%)
ALT (U/L)	41.2 ± 19.6
AST (U/L)	39.8 ± 17.2
ALP (U/L)	122.4 ± 58.7
Total bilirubin (mg/dL)	0.88 ± 0.34

**Table 2:** MRCP-Based Radiologic Findings in the PCS Cohort.

Parameter	PCS Group (n = 141), n (%)
CBD diameter ≥7 mm (dilated)	45 (31.9%)
Biliary dilatation (any degree)	21 (14.9%)
Biliary stricture	4 (2.8%)
Oddi sphincter dysfunction	16 (11.3%)
Postoperative radiologic complications (biloma, abscess, collection)	56 (39.7%)

CBD diameter and ROC analyses were restricted to the MRCP subgroup (n = 45).

**Table 3:** Endoscopic Findings of the PCS Group (n = 141).

Parameter	PCS Group (n = 141), n (%)
Mucosal inflammation (gastritis, duodenitis, ulcer)	129 (91.5%)
Bile reflux	18 (12.8%)
Papilla Vateri edema/hyperemia	11 (7.8%)
Oddi sphincter dysfunction (signs on endoscopy)	16 (11.3%)
Combined mucosal + papillary pathology	9 (6.4%)
Normal endoscopic findings	12 (8.5%)

**Table 4:** Correlation between Radiologic and Endoscopic Findings.

Variable Pair	Correlation Coefficient (r)	*p*-Value
CBD diameter vs. mucosal inflammation	0.32	0.001
Biliary dilatation vs. bile reflux	0.28	0.004
Oddi sphincter dysfunction vs. papillary edema/hyperemia	0.41	0.001
Biliary stricture vs. papillary deformity	0.22	0.03
Postoperative complications vs. mucosal inflammation	0.18	0.06

**Table 5:** ROC Analysis Results for MRCP-Based Predictors of Biliary Dilatation/Abnormal Biliary Findings.

Parameter	AUC (95% CI)	Cut-Off Value	Sensitivity (%)	Specificity (%)	*p*-Value
CBD diameter (mm)	0.82 (0.74–0.90)	≥8.0	78.3	81.5	<0.001
Biliary dilatation (any degree)	0.76 (0.67–0.85)	–	72.4	70.3	0.002
Oddi sphincter dysfunction	0.69 (0.58–0.80)	–	63.5	71.4	0.011
Biliary stricture	0.62 (0.51–0.73)	–	55.6	68.0	0.046

**Table 6:** Multivariate Logistic Regression Analysis: Predictors of Biliary Dilatation (n = 141).

Variable	OR	95% CI	*p*-Value
Age (per year)	1.05	1.01–1.09	0.007
Male (vs. Female)	1.00	0.34–2.94	0.994
Oddi sphincter dysfunction	0.37	0.04–3.14	0.359
Biliary stricture	9.12	0.78–106.08	0.077
Postoperative complications (any)	1.00	0.35–2.84	0.994
Intercept	0.01	0.00–0.10	<0.001

## Data Availability

The datasets used and/or analyzed during the current study are available from the corresponding author upon reasonable request.

## Abbreviations

ALT	Alanine aminotransferase
AST	Aspartate aminotransferase
AUC	Area under the (ROC) curve
CBD	Common bile duct
CI	Confidence interval
CT	Computed tomography
EGD	Esophagogastroduodenoscopy (upper GI endoscopy)
ERCP	Endoscopic retrograde cholangiopancreatography
IQR	Interquartile range
MRCP	Magnetic resonance cholangiopancreatography
OR	Odds ratio
PCS	Post-cholecystectomy syndrome
ROC	Receiver operating characteristic
SD	Standard deviation
SOD	Sphincter of Oddi dysfunction
US	Ultrasonography
